# The Shouldice technique for the treatment of inguinal hernia

**DOI:** 10.4103/0972-9941.27723

**Published:** 2006-09

**Authors:** Chin Keung Chan, Gabriel Chan

**Affiliations:** Shouldice Hospital, 7750 Bayview Avenue, Thornhill, Ontario L3T 4A3, Canada

**Keywords:** Inguinal hernia, Shouldice repair, recurrence

## Abstract

The Shouldice repair has been refined over several decades and is the gold standard for the prosthesisfree treatment of inguinal hernias. A recurrence rate around 1% has been consistently demonstrated over the years. The objective of this paper is to outline and highlight the key principles, including the dedicated pre-operative preparation, the use of local anesthesia, a complete inguinal dissection and the eponymous four-layered reconstruction. A knowledge and understanding of inguinal hernia anatomy and the patho-physiology of recurrence are vital to achieving a long-term success and patient satisfaction for a pure tissue repair.

The Shouldice hospital has specialized in the care of abdominal wall hernias for more than 60 years. Currently 7,500 patients are treated every year. The Shouldice technique is a well-established operation for both primary and recurrent inguinal hernias.[1] A recurrence rate around 1% has been demonstrated by our group and many others over the years.[2–5] The principle of the technique is based on more than the technical aspect alone and includes the preoperative preparation, anesthesia, comprehensive inguinal dissection and postoperative principles.

## Preoperative care

The comprehensive care of an inguinal hernia begins with the initial office consultation. Interview should include a detailed history of symptoms, which are typically mild discomfort and a lump. One scenario that warrants care is pain and symptoms which are incongruent with such a small or ‘occult’ hernia. In particular, pain with a constant quality and persistence in the recumbent position are approached with caution. Physical examination should document the hernia, noting the size and location. A differentiation should be made between an inguinal and femoral hernia, based on the position relative to the inguinal ligament. All patients are counseled regarding weight reduction and exercise regimens, if necessary. An extended waiting period can be used to achieve weight targets; however, if symptoms are significant enough to prevent activities of daily living, a more urgent schedule can be adopted.

## Anesthesia

Operations at the Shouldice hospital are performed with local anesthesia. Preoperative sedation, 1 h prior to entering the operating theater, consists of intramuscular meperidine and oral diazepam. Local subcutaneous infiltration of 1% procaine hydrochloride begins 2 cm medial and inferior to the anterior superior iliac spine and proceeds to the pubic tubercle. The clinical benefits of local anesthesia extend beyond an avoidance of a general anesthesia. It allows the cooperation of the patient to strain upon request to aid in the search for occult hernias. In addition, with a normal abdominal wall muscle tone, the surgeon is able to accurately judge the amount of tension created by the reconstruction.

Further infiltration is also required during various stages of the inguinal dissection. The inguinal canal is infiltrated to anesthetize the ilio-inguinal and ilio-hypogastric nerves prior to opening the inguinal canal. The internal oblique, superior to the canal is also infiltrated. Later, upon mobilization of the spermatic cord, the internal ring, posterior floor and cremasteric muscle are infiltrated to anesthetize the genital branch of the genital-femoral nerve, found usually traveling within the cremasteric muscle fibers, the peritoneum of the indirect sac and the vas deferens. If further conscious sedation is required during the procedure, midazolam and fentanyl can be administered.

General anesthesia is generally not required. We reserve this for operations on multiple recurrent hernias, where local infiltration of scar tissue is unlikely to provide adequate anesthesia.

## Inguinal dissection

A comprehensive intraoperative examination of the inguinal region is performed in all cases. This is integral to attaining a very low recurrence rate. The frequency of secondary hernias found at the time of surgery is 15.4%.[6] The assessment should include the direct, indirect, interstitial and femoral spaces.

The incision is made from 2 cm inferio-medial to the ASIS to the pubic tubercle, parallel to the inguinal ligament. This allows generous exposure to both inguinal canal and femoral space. Trendelenburg's position may be used to reduce intra-abdominal pressure, facilitating the repair.

Dissection is deepened to expose the external oblique fascia and superficial inguinal ring. Local anesthesia is required at this point prior to opening the canal. Care is required to identify and avoid injury to the ilio-inguinal and ilio-hypogastric nerves. Their course usually runs deep to the external oblique, superior to the deep ring and superficial to the cord structures. Infiltration of the internal oblique muscle superior to the canal can provide anesthesia for the internal oblique and peritoneum used later in the reconstruction. If the nerves are traumatized at any point, we do not hesitate to resect the damaged segments to avoid neuropathy postoperatively. The resultant numbness after nerve resection is not usually of any clinical significance.

**Figure 1:**
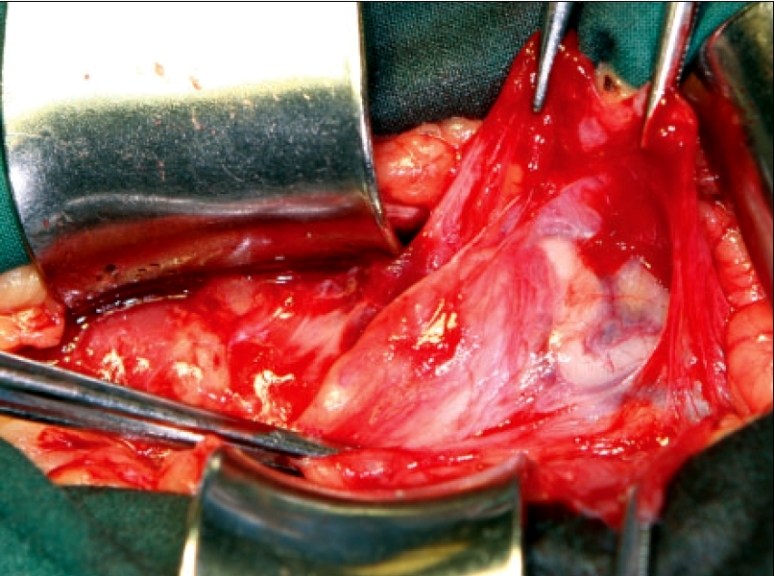
Right inguinal hernia repair: dissection of cremasteric muscle. Mobilization of the spermatic cord through the cremasteric muscle and creation of medial and lateral flaps for transection

The spermatic cord is isolated by a longitudinal anterior opening in the cremasteric muscle fibers at the midpoint of the canal extended medially to the pubic tubercle, creating two flaps of muscle medial and lateral to the cord [Figure 1]. This maneuver allows a complete examination of the posterior floor, in particular the medial aspect. The larger lateral cremasteric flap, which includes the genital branch, is infiltrated with local anesthetic, as is the internal ring. If a lipoma is present, this is dissected from the cord towards the internal ring and excised. This facilitates identification of the indirect sac in its typical antero-medial position. An indirect sac should be isolated from the cord and internal ring and excised. The stump should retract naturally into the peritoneal cavity. Some surgeons choose only to reduce the sac to avoid peritoneal irritation and discomfort. Care should be taken if the sac is thicker, contains more fat than usual or is broad based, as this may be an indication of a sliding hernia. These are reduced and not opened. If a sac is not identified, the dissection of the cord at the internal ring must identify a peritoneal protrusion, which can be aided by gentle traction on the cord. The identification of this structure, which is in its normal anatomic position, assures the surgeon that the indirect hernia has not been missed. After complete dissection of the cord, both flaps of the cremasteric muscle are divided sharply and ligated. These stumps are used to support the repair at a later point in the procedure.

**Figure 2:**
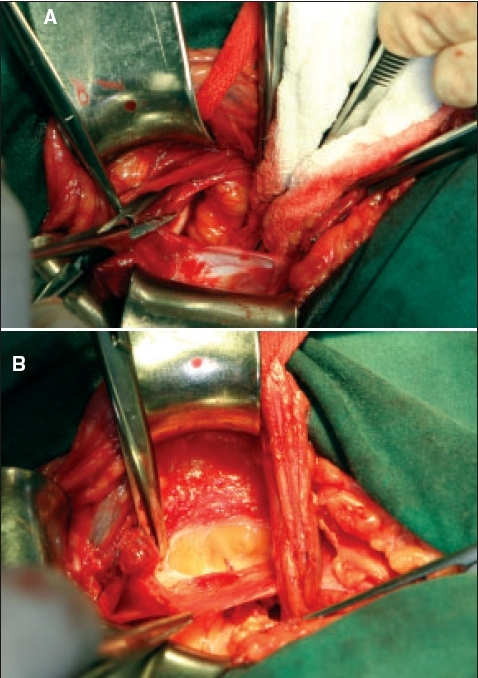
Dissection of femoral space A) Cooper's ligament seen through the preperitoneal space, deep to the tips of the clamp; and B) Incision of the cribiformis ligament below the inguinal ligament

The posterior wall of the inguinal canal is examined for a direct hernia then opened beginning at the internal ring in parallel to the internal oblique muscle fibers. Care should be taken not to injure the inferior epigastric vessels found medial to the internal ring. The lateral flap should be wide enough, at least 1 cm, to reach the edge of the rectus sheath in the first layer of the repair. Any redundant transversalis fascia of a direct hernia should be excised. Preperitoneal examination of the internal oblique muscle and fascia cephalad to the internal ring and the inguinal canal, should confirm the presence of an interstitial hernia. If present, the internal ring can be incised laterally to include an interstitial defect. The internal ring would then be displaced laterally after reconstruction. Dissection below the inguinal ligament should identify Cooper's ligament and thus confirm the presence or absence of a femoral hernia [Figure 2A]. The superficial thigh fascia or cribiformis fascia caudal to the inguinal ligament should be incised to examine the femoral space from below [Figure 2B]. In addition, this mobilizes the inguinal ligament and external oblique for use in the reconstruction.

## Reconstruction

A continuous repair with 32–34 gauge stainless steel wire is used for the reconstruction. This may be substituted with a 3–0 polypropylene suture. It is a four-layer tissue reconstruction using two separate sutures. A continuous technique distributes the strength of the repair evenly and should be without tension. A relaxing incision is rarely required.

**Figure 3:**
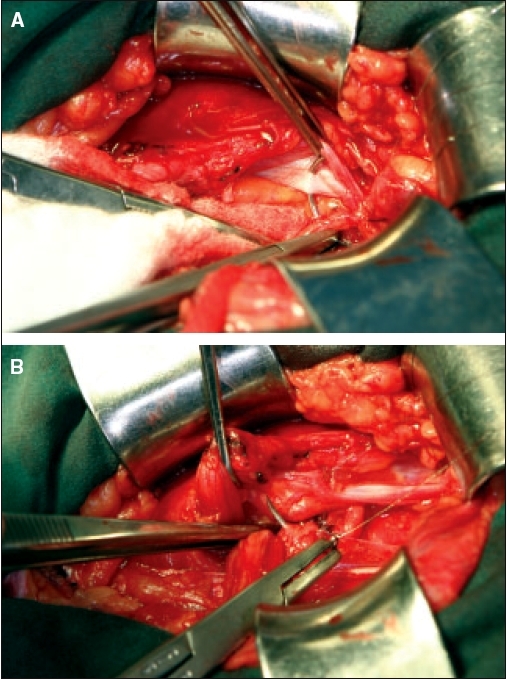
Reconstruction: the first layer. A) Starting at the pubic tubercle, the lateral flap of transversalis fascia is taken to the edge of rectus sheath underneath the medial flap; and B) The layer is completed with the reconstruction of internal ring. The lateral stump of cremasteric muscle is taken with the bite of transversalis to buttress its medial edge of the new internal ring, prior to emerging with a full thickness bite of internal oblique.

**Figure 4:**
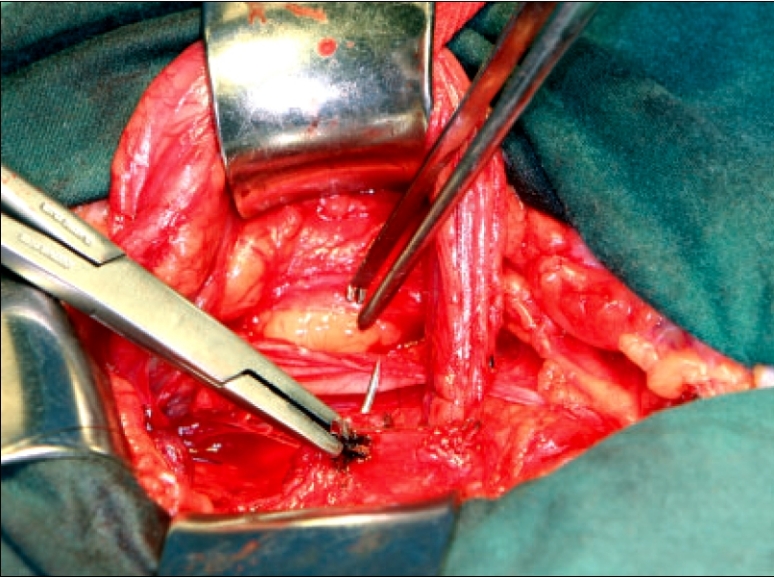
Reconstruction: the second layer Using the same suture, it is continued from the internal ring back, taking the medial flap of transversalis fascia to the shelving portion of the inguinal ligament. Overlapping layers are created.

The first two layers represent an overlapped reconstruction. It begins medially, anchoring over the pubic tubercle, leaving a sufficient end to tie the returning suture after the second layer. The inferolateral flap of transversalis fascia is sutured to the lateral edge of the rectus sheath by reaching underneath the superior-medial flap [Figure 3A]. The reconstruction then moves laterally to the aponeurosis of the transversus abdominis and the edge of the internal oblique muscle. The lateral extent of this layer redefines the internal ring and should include the superior stump of the divided lateral flap of cremasteric muscle [Figure 3B]. This buttresses the internal ring and helps prevent an indirect recurrence. The suturing is then reversed to begin the second layer. The superior flap of transversalis fascia is sutured to the shelving portion of the inguinal ligament then tied at the pubic tubercle [Figure 4]. The periosteum should not be included in any bite as this can result in a painful osteitis.

**Figure 5:**
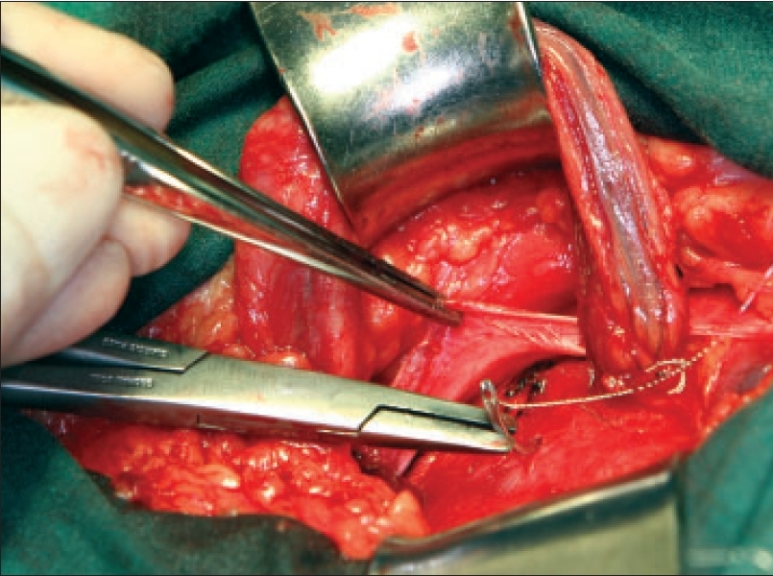
Reconstruction: the third layer. Starting at the medial side of the internal ring, the external and internal oblique are used to imbricate the first two layers. Small bites of external oblique are taken just above the inguinal ligament.

**Figure 6:**
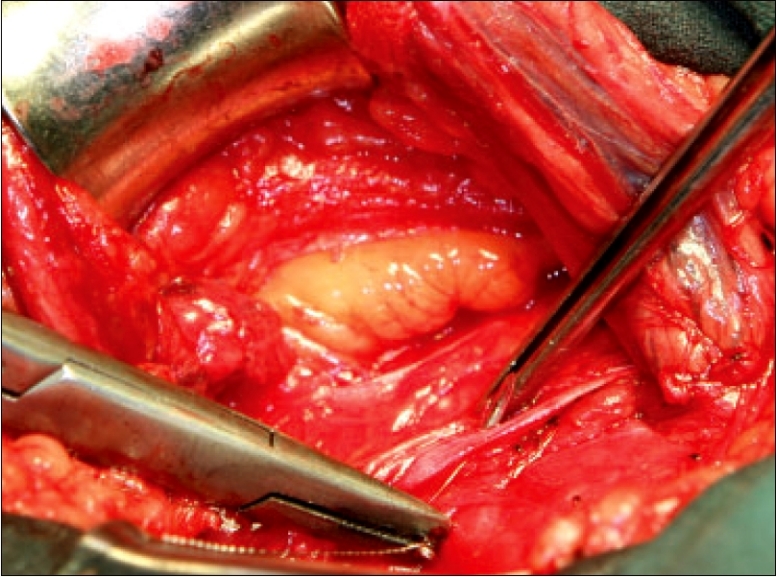
Reconstruction: the fourth layer. The second layer of imbrication using the external and internal oblique. The benefit of incising the cribiformis fascia and the mobilization of the lateral flap of external oblique is realized as a small flap of external oblique remains after the four layers. This small flap is used to reconstruct the inguinal canal and restore the natural anatomic position of the cord structures.

The next stage of reconstruction creates a two-layered imbrication to provide reinforcement. The layer is begun superior and slightly lateral to deep ring, anchoring the suture to the internal oblique fascia. The inferior flap of the external oblique, millimeters above and parallel to the inguinal ligament, is tacked to the edge of the internal oblique and transversus muscles [Figure 5]. In addition, only a small bite of the internal oblique is required, no more than 5 mm. Excessively large bites will create tension. At the pubic tubercle, the direction is reversed for the fourth layer and taken back to the internal ring and affixed [Figure 6]. The importance of mobilizing the inferior flap of external oblique by previously incising the superficial thigh fascia is realized here. The additional mobility allows the external oblique to be used in the third and fourth layers to cover the medial portion of the repair that is susceptible to recurrence. The inguinal canal is reconstructed by re-approximating the remaining external oblique fascia, returning the cord to its anatomical position. The inferior stump of the medial flap of cremasteric muscle is included in the first suture medially to stabilize the position of the testes to the abdominal wall and prevent drooping.

## Postoperative course

In the postoperative care, oral narcotic analgesia is usually required during the first 48 h. Ambulation is encouraged the evening after the operation and light exercises commence the first postoperative day. Upon discharge, heavy lifting can be resumed at 4 weeks.

## CONCLUSION

The Shouldice technique is a comprehensive treatment plan for inguinal hernias based on the extensive experience at the Shouldice Hospital. Thorough knowledge of inguinal anatomy and pathology is essential to achieving optimal results. Addressing all factors involved in hernia development and recurrences should be incorporated into the routine care of all patients with inguinal hernias. Outcomes of this pure tissue repair and ultimately patient satisfaction, depend on this.
